# C-853-06. Improving Splint Adherence in Burn Patients

**DOI:** 10.1093/jbcr/irag033.074

**Published:** 2026-04-06

**Authors:** Annalene Arellano, Michael Nunez, Preeti Gulati, Tino Hardy, Clifford C Sheckter

**Affiliations:** Santa Clara Valley Medical Center Regional Burn Center, San Jose, CA; Santa Clara Valley Medical Center, San Jose, CA; Santa Clara Valley Medical Center Regional Burn Center, San Jose, CA; Santa Clara Valley Healthcare System, San Jose, CA; Stanford University School of Medicine, Stanford, CA

## Abstract

**Introduction:**

Joint splinting is standard in caring for burn patients to preserve range of motion and facilitate graft take. Splint adherence is particularly challenging in patients with delirium, agitation, and young age which can lead to suboptimal outcomes. A burn physical therapy (PT) and occupational team (OT) aimed to systematically improve adult and pediatric splint adherence leveraging implementation science.

**Methods:**

Using the Consolidated Framework for Implementation Research, a verified burn center created a splint adherence bundle. 37 practitioners were interviewed across seven domains (3 OTs, 5 PTs, 2 burn surgeons, 16 patients, 1 social worker, 1 psychologist, 9 nurses). Four patient factors (knowledge, health literacy, agitation/delirium, and self-advocacy) and five system factors (splint design, nursing staff knowledge, nursing staff turnover, inter-therapist communication, and time for splint application) were identified through thematic analysis which informed the bundle. The bundle underwent iterative changes during the study period. Phase 1 interventions partnered nursing and therapy with communication on a white board, sticky notes in electronic medical record, daily reminders on rounds, and nursing education during orientation. Phase 2 interventions included improving pharmacologic pain and delirium management, splinting discussions in psychology therapy sessions, and family engagement with splint re-application. The primary outcome was splint adherence as measured by PT/OT at two separate times per day. Non-adherence was defined as not wearing the splint or finding the splint applied in incorrectly. Significance was determined using the Fisher exact test with alpha of 0.05.

**Results:**

Baseline splint adherence was 57% for adults and 67% for pediatric patients. After bundle implementation, 40 adult patients and 10 pediatric patients were evaluated over a one-year period yielding 1649 adult observations and 555 pediatric observations. Splint adherence improved in adult patients (72%) after phase 1 and marginally improved in pediatric patients (68%). After phase 2 implementation, adult splint adherence was 86% (29% increase, *p*<.01), and 92% in children (25% increase, *p*<.01).

**Conclusions:**

Splint adherence was improved using an implementation science framework. Splinting non-adherence was largely influenced by gaps in patient and nursing knowledge along with inadequate pharmacologic management of pain and delirium. The multifaced bundled improved splint adherence for both children and adults.

**Applicability of Research to Practice:**

Burn centers experiencing challenges with splint adherence may consider adopting and/or modifying a multifaceted bundle that has been shown to improve adherence.

**Funding for the Study:**

N/A.

